# Detection of Periodic Leg Movements by Machine Learning Methods Using Polysomnographic Parameters Other Than Leg Electromyography

**DOI:** 10.1155/2016/2041467

**Published:** 2016-04-24

**Authors:** İlhan Umut, Güven Çentik

**Affiliations:** Department of Computer Engineering, Faculty of Engineering, Trakya University, 22030 Edirne, Turkey

## Abstract

The number of channels used for polysomnographic recording frequently causes difficulties for patients because of the many cables connected. Also, it increases the risk of having troubles during recording process and increases the storage volume. In this study, it is intended to detect periodic leg movement (PLM) in sleep with the use of the channels except leg electromyography (EMG) by analysing polysomnography (PSG) data with digital signal processing (DSP) and machine learning methods. PSG records of 153 patients of different ages and genders with PLM disorder diagnosis were examined retrospectively. A novel software was developed for the analysis of PSG records. The software utilizes the machine learning algorithms, statistical methods, and DSP methods. In order to classify PLM, popular machine learning methods (multilayer perceptron, *K*-nearest neighbour, and random forests) and logistic regression were used. Comparison of classified results showed that while *K*-nearest neighbour classification algorithm had higher average classification rate (91.87%) and lower average classification error value (RMSE = 0.2850), multilayer perceptron algorithm had the lowest average classification rate (83.29%) and the highest average classification error value (RMSE = 0.3705). Results showed that PLM can be classified with high accuracy (91.87%) without leg EMG record being present.

## 1. Introduction

Since the 1950s, sleep disorders have become a field of expertise in which approximately 90 different diseases have been described [1]. Polysomnography (PSG) is still one of the most effective methods in the diagnosis of the sleep diseases. This method is based on simultaneous multichannel recording of body signals during sleep. The number of channels used for polysomnographic recording frequently causes difficulties for patients because of the many cables connected during their sleep. On the other hand, it increases the risk of having troubles during recording process and affects the storage volume in a negative way. In PSG, leg electromyography channels are used to record leg movements; recording leg movements is of great importance to diagnose periodic leg movements during sleep.

Periodic limb movement disorder (PLMD) is a sleep disorder in which leg movements recurs periodically during sleep and is formed with quite stereotypical foot, leg, and/or arm movements. PLMD has negative effects such as excessive sleepiness in the daytime, waking up at night, disorders in sleep cycle, frequently waking from sleep, and kicking your bed partner [2]. Leg electromyography markings from PSG records are used for diagnosis of PLMD along with other criteria. PLMD scoring and the polysomnographic evaluation were standardized in 1993. These criteria have still been accepted as the golden standard in PLMD researches [3]. The occurrence frequency of periodic leg movement (PLM) or hourly PLM count is used to determine PLM index. Normal value of this index should be below 15 PLMs per hour [4].

In this study, we investigated an alternative method to detect leg movements without using leg EMG channels in PSG. And then, in order to test the hypothesis that leg movements can be detected without using leg EMG, data from 153 real patients were used and 768,726 epochs were analysed.

## 2. Material and Methods

### 2.1. Data Collection

The selection and preparation of the data in the study prove to be an important step in data mining applications [5]. After the hypothesis was determined, PSG records of 153 patients of different ages and genders (male/female, 112/41) with PLMD diagnosis were examined retrospectively. Leg movements can be seen as a consequence of breathing disorders during sleep. Towards end of an apnea period, leg movement can be observed as a reflexive response to hypoxia and awakening. Leg movements can also be observed as a separate event, independent of apnea periods. Thus, we exclude leg movements accompanying an apnea period and focus solely on independent leg movements. General characteristics of study group are shown in Table 1.

All PSG recordings were performed by using a 44-channel PSG device (Compumedics 44E series, Australia). Each record was approximately 7 to 8 hours. Sleep scoring and event scoring were made manually by a sleep specialist, as the reliability of automatic scoring systems is low. PSG assembly involves 6-channel EEG (F3-A2, F4-A1, O1-A2, O2-A1, C3-A2, and C4-A1), right and left electrooculography (EOG) (LOC-A2 and ROC-A1), leg electromyography (EMG), chin EMG, electrocardiography (ECG), oxygen saturation with fingertip pulse oximeter (SpO_2_), thermistor (for upper airway signals), thorax and abdomen, snoring (microphone), and measurement of body positions. EEG electrodes were placed according to the internationally accepted 10–20 system [6]. It was determined that the sampling rate used during the recording of lung and upper airway signals is 256 Hz, the sampling rate for thoracic and abdominal respiration signals is 128 Hz, and the lower and upper frequencies of the filtering done are 0.3 Hz and 30 Hz, respectively.

PSG data were scored according to the international classification of disorders produced by American Academy of Sleep Medicine [7] and Rechtshaffen and Kales scoring manual criteria [8]. The scoring criteria of PLMD were first proposed by Coleman in 1982. In the study, PLM is defined as follows: movements should last between 0.5 sec and 5 sec. The activity should be 8 millivolts above the resting EMG amplitude. There should be 4 or 5 movements occurring successively. The time between movements (the time passing from the beginning of a movement until the beginning of another movement) should not be shorter than 5 sec and longer than 90 sec [9].

### 2.2. Data Filtering

Obtaining clean and error-free data for the classification is one of the crucial factors which affect results. Therefore, all the data were exposed to a selection operation before classification.

Some errors may occur, though rarely seen, during the automatic scoring operation. These automatic scorings are generally verified by a specialist, yet sometimes these errors are also overlooked by the specialists. As there are lots of channels and the main focus of specialists is on apnea scorings, leg movements errors can be overlooked with more frequency. In addition, there are artefacts in some epochs of PSG records. These artefacts result from patients' bodies (eye movements, ECG) and external factors (instrumental, electrode). These artefacts can also cause noise in other channels of the PSG record. The artefacts formed on any of the channels, especially when they are densely formed, can damage the natural structure of the signals in channels, which affects the result of any analysis applied in a negative way. A more sophisticated method of detection and removal of artefacts may minimize the negative effects caused by the artefacts.

In our study, the first and the most important one of these operations is that the undesired artefacts in all the signals in PSG records were eliminated by manual scoring instead of automatically. In situations where it was detected that there was an artefact in at least one signal in an epoch, that epoch was not used in the classification. This caused a great number of epochs to be eliminated as part of selection operation before classification. Generally, during the apnea or towards the end of apnea, leg movements stemming from apnea occur. As the second step, the epochs where leg movements resulting from apnea exist were eliminated in order to separate apnea from PLM. The exclusion rule of software was that if there was apnea, this epoch was excluded together with 15-second segment before and 15-second segment after this apnea from the analyses. In our case, a leg movement segment was at least 15 seconds far from any respiratory event. And the last elimination operation was conducted in the epochs which occurred during wakefulness period of PSG records. After all these three elimination stages, 275,865 of 768,726 epochs were removed. At the end of removal operation, there were 492,861 epochs left. Of all these epochs, 46,020 epochs were with PLM, and 446,841 were without PLM.

Following the removal operation, resample filtering operation was carried out in order to balance huge and unbalanced data and increase the learning performance [10]. The number of epochs with PLM was equated with the number of epochs without PLM. 446,841 samples without PLM were balanced to 46,020 samples with PLM, leading around 46,000 samples for each case. This reduction process was made manually in order to make sure that each and every patient record from 153 patients was equally represented in the data to be analysed. In the analysis conducted, various sample size percent values were compared for performance improvements. It was observed that the accuracy decreases in the values below 10. The tests were carried out with *K*-nearest neighbor (*K*-NN), which gives the highest classification value. At the end of these analyses, the sample size percent value for accuracy and performance was chosen to be 10%. With selection of 10% as sample size percent value, the number of 46,000 samples for each case is reduced to around 4.600 samples for each case, 4602 epochs with PLM and 4,684 epochs without PLM to be precise. This reselection sampling was also applied patient-wise, sampling each patient's record randomly to have data points randomly distributed in full recording. This was aimed at making sure that every patient is represented and no fixed period (i.e., first hour of sleep) in the record is biased. Besides, no random values were produced; only recorded values were used.

At the end of the operations conducted, 9.286 epochs were used for the classification and 4.602 of these epochs were with PLM and 4.684 of them were without PLM.

### 2.3. Features

**Figure 1:**
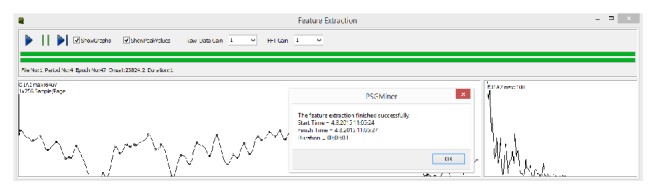
The graphical user interface of the feature extraction module.

Being the minimum value for PLM interval, 5 seconds was chosen as the epoch length. PSG record segments were taken from all channels except leg EMG channels and were analysed through a software which uses digital signal processing methods, developed by our team [11]. The graphical user interface of the feature extraction module is shown in Figure 1. The specific attributes of the signals presented in PSG record was obtained for each epoch. Each record of epoch attributes was stored in MySQL database with an ID value created through the use of patient number and epoch number.

It is possible to achieve different attributes from EEG [12], ECG [13], chin EMG [14], and other signals. However, as different channels have different attributes for PSG channels, not all the attributes were used in the study. The attribute evaluation operation was carried out for all PSG channels and the attributes producing the classification with the highest gain were selected.

DSP, statistical methods, raw data (in EDF file), and scoring values (in XML files) were used in achieving the attributes, and 77 attributes achieved from different signals were used in the classification of PLMs. The attributes used in the classification are shown in Table 2.

Firstly, Haar wavelet transformation technique was implemented in obtaining the subfrequency bands of EEG signals: delta (*δ*) (0.5–4 Hz), theta (*θ*) (4–8 Hz), alpha (*α*) (8–13 Hz), and beta (*β*) (13–30 Hz) [15]. Among various wavelet transformation methods, we chose Haar wavelet transformation as it is the simplest and shortest way of transformation [16].

Secondly, Discrete Fourier Transform (DFT) was used to obtain the spectrum values of subfrequency bands [17]. With this technique, PSG signals that are in the time domain were transformed into the frequency domain. DFT was also used on ECG and chin EMG channels to obtain mean power spectrum and spectral entropy values for these channels.

Finally, obtaining all attributes (N: 466), the attributes were evaluated according to their gain ratios. In the evaluation operation, GainRatioAttributeEval method was used to calculate ranker parameter value for each attribute. This method evaluates how good an attribute is through measuring the gain ratio related to the class:

(1) GainRClass,Attribute=HClass−HClass ∣ AttributeHAttribute,

where *H* is the entropy.

At the end of the attribute evaluation operation, the first five signal attributes yielding the highest classification gain are the attributes which were taken from SpO_2_ (ranked: 0.04126), C3-A2 (ranked: 0.02997), chin EMG (ranked: 0.02584), F3-A2 (ranked: 0.01825), and O1-A2 (ranked: 0.01812) signals. The results indicated that attributes of “SpO_2_” provided the most predictive estimation.

### 2.4. Classification Methods

**Figure 2:**
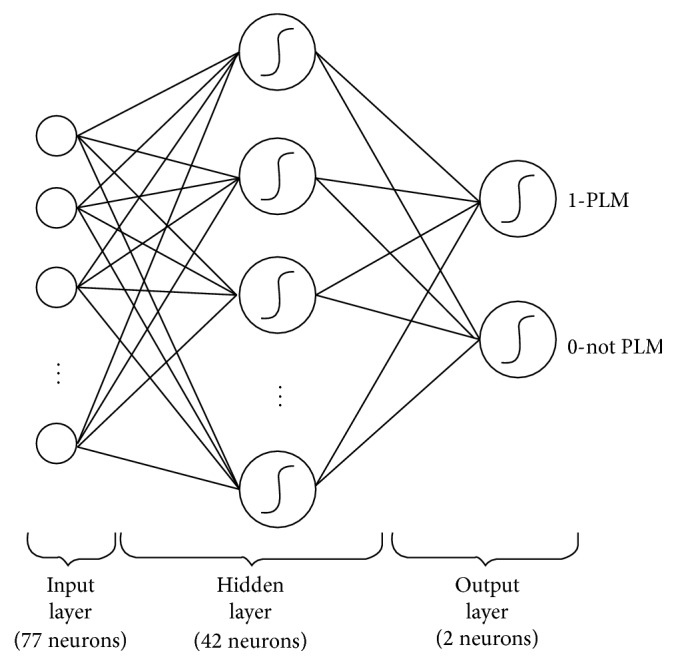
Graphical representation of the MLP model.

It is possible to put data mining techniques in 5 main groups. These are classification, clustering, regression models, association rule, and sequential patterns. The methods of data mining which are commonly used for making guesses are classification and regression models [18]. We used four different types of classification models: multilayer perceptron (MLP) (parameters: Learning Rate = 0.3, Momentum = 0.2, Iteration = 500, Hidden Layer Count = 1, Hidden Layer Neuron Count = 42, and Activation Function = Sigmoid) [19], random forests (parameters: Trees Count = 100) [20], *K*-NN (parameters: *K* = 1) [21], and logistic regression (parameters: Ridge = 1.0*E* − 8) [22]. These models were used in this study because they have been highly used in recent literature and they performed really well in preliminary comparative studies [23].  Figure 2 shows the graphical representation of the MLP architecture used in this study.

## 3. Results

While the model was being developed, a certain amount of the data were used for training, and the remaining data were used for testing. Using the WEKA classification algorithms, approximate operations of the test data classes were carried out. The performance comparisons of machine learning classification algorithms were made with the values of accuracy, RMSE and confusion matrix. 10-fold cross-validation technique was employed for all the classifiers as a test option [24]. In this method, the data cluster is divided into 10 equal pieces and 1 of the ten equal pieces is used for the test and the remaining 9 pieces are used for training. According to this, 8357 epochs were used for training and 929 epochs were used for the test. The test data was tested with the classification algorithms existing in WEKA on 10-fold cross-validation data cluster.

Trial-and-error procedure was implemented for a number of nearest neighbors, *K*, ranging from 1 to 10. The improvement of the performance with an increasing number of nearest neighbors is less noticeable for more than 7 neighbors and there is no marginal improvement in the overall performance when increasing *K* beyond 7. Small values (from 1 to 4) of the feature dimension gave the most satisfactory results for each given number of neighbor vectors *K*.

Parameters for other algorithms were tested with similar procedures and optimum values for each parameter were obtained experimentally for each algorithm.

The algorithms used in machine learning were repeated for 10 times using the same datasets. 100 results were obtained by using 10-fold for 10 times for a classification method, and 400 results in total were obtained for all classification methods. The average and standard deviation values of all methods are shown in detail for each fold in Table 3.

According to the results obtained on the basis of average classification success percentage, while *K*-NN classification algorithm has a higher classification rate (91.87%) and a lower error value (RMSE = 0.2850), MLP algorithm has the lowest classification rate (83.29%) and the highest error value (RMSE = 0.3705).

The results show that these two algorithms (*K*-nearest neighbor and decision tree algorithms) have a very high capacity of classification and are powerful. In fact, they are so effective that unless there is class ambiguity they can provide perfect classification on the training data no matter how many instances are given, even for very high number of instances. This, of course, means a high probability of fitting noisy instances and outliers, that is, overfitting.

*K*-NN method can do simple calculations. It can also adapt both intraclass and interclass changes due to age, gender, weight, and so forth and as a result can be used with linearly inseparable problems. It is known that *K*-NN classifier gives better results with least redundant features [25]. In Table 3, it is shown that, compared to others, the errors in confusion matrix are balanced for both classes. MLP, as there is random initialization, does not always perform best fit to data and it can stuck at local minima, which does not lead to an optimum solution (result). As a result of all these factors, *K*-NN performed much better than other methods, especially compared to MLP. Random forests method was successful, possibly due to random selection, which can eliminate unnecessary features [26].

## 4. Discussion

Our study has three significant features. Generally, statistical methods are used to identify PLM. First, as a novel approach, we applied artificial intelligence methodology to find PLM. Second, a software is developed for this study, utilizing digital signal processing and machine learning methods. And for the first time we used this software to analyse electrophysiological recordings of patients with sleep disorder. Third, we used quite a big sample space of 768,726 events to determine the PSG recordings in classifying periodic leg movements during sleep.

Through the use of *K*-NN classification, one of the machine learning methods, PLMs were correctly classified with the ratio of 91.87%. During this operation, leg EMG signals were not employed. PSG recording requires many electrodes and sensors on human body from head to feet. Reducing these connections may provide a more comfortable test for both patient and technician. If PLMs can be defined through the signals obtained from other sources than those placed in the right or left leg, significant savings would be made in PSG recording.

The sensors and electrodes, from which the signals will be received during the recording, are placed on the patient's body before PSG recording by a sleep technician. This operation takes approximately 45–50 minutes, and it is a demanding job to place the electrodes. If the number of electrodes and sensors to be placed on a patient's body is decreased, thus, the workload of technician is reduced. Additionally, the time spent on placing electrodes and sensors is shortened, making the waiting time of patients shorter as well. It also increases patient comfort during sleep, as it is quite uncomfortable to try to sleep entangled with many wires.

Also, patients can move more freely due to the reduction in number of these electrodes and sensors. The electrodes placed on the right and left legs are recorded on the computer with 128 samples per second. Considering that these two electrodes were not used, the storage unit would be used in a more economical way, for the number of data to be recorded on these storage units would decrease by 15%.

Scoring and reporting a PSG recording is a labour-intensive process, which requires several hours. Automatic scoring systems are not efficient and laboratory staff mostly does PSG reporting manually. A decrease in number of channels in PSG may save time and help manual scoring. Four pieces of cable and 2 electrodes are used for two leg EMGs in PSG record. If the channels of leg EMG are not used, big savings can be made on cable and electrodes; thus, the cost of PSG is brought down.

The results indicated that the predictive signal “SpO_2_” is by far the most important predictor. Why these prognostic factors are more important predictors than the others is a question that can only be answered by medical professionals and further clinical studies.

Although machine learning methods are capable of extracting features hidden deep into large medical datasets, without the collaboration from the medical professionals, their results are useless. The features found via machine learning methods should be evaluated by medical professionals who have expertise in sleep. Data mining is not aiming to replace medical professionals and researchers but to complement their invaluable efforts to save more human lives and to save their valuable time.

In short, the study has strong sides. In the literature search carried, no other study on this subject using this methodology was found. Also, advanced statistical methods were employed in obtaining the results. Data from 153 real patients were used in the study and 768,726 cases were analysed for the patients. The use of high amount of data enabled us to obtain more meaningful and statistically robust results as well. A software is developed for this study, employing techniques discussed here along with many other features and it will be offered to the community to aid them in their work.

## Figures and Tables

**Table 1:** General characteristics of the study group.

	Mean ± SD	Range
Age	61.48 ± 11.54	32–94
Gender, M/F, number	112/41	73%/27%
Weight, kg	93.92 ± 20.19	47–195
Height, cm	170.90 ± 8.57	150–192
ESS	17.59 ± 28.40	0.5–247.5
Time in bed, min	419.85 ± 53.08	189.80–545.40
Total sleep time, min	343.03 ± 73.19	60.5–475
Sleep efficiency, %	82 ± 14	29–98
N1, % of SPT	6 ± 4	1–33
N2, % of SPT	63 ± 14	29–94
N3, % of SPT	18 ± 12	0–55
REM, % of SPT	14 ± 7	0–38
AHI	5.19 ± 7.54	0–77.3
Total number of apnea	25.63 ± 19.92	0–101
Total number of obstructive apnea	23 ± 17.7	0–71
Total number of mixed apnea index	1.97 ± 3.78	0–23
Total number of hypopnea	19.58 ± 25.95	0–129
RDI	8.56 ± 8.75	0–66.7
REM RDI	8.92 ± 9.74	0–50.7
non-REM RDI	8.21 ± 9.01	0–66.7
Minimum oxygen saturation	84.88 ± 7.27	64–100
Saturation between 81% and 90%, min	40.80 ± 54.62	0–278.5
Number of leg movements/hour of sleep	66.86 ± 16.26	28–100

AHI, apnea-hypopnea index; ESS, Epworth Sleepiness Scale; F, female; M, male; SD, standard deviation; SPT, sleep period time; RDI, Respiratory Disturbance Index.

**Table 2:** The attributes used in the classification.

Number	Signal	Feature
1	C3-A2	Delta
2	C3-A2	Theta
3	C3-A2	Alpha
4	C3-A2	Beta
5	C3-A2	Zero crossings
6	C3-A2	Mean
7	C3-A2	Mean power spectrum
8	C3-A2	RMS
9	C3-A2	Spectral entropy
10	C4-A1	Delta
11	C4-A1	Theta
12	C4-A1	Alpha
13	C4-A1	Beta
14	C4-A1	Zero crossings
15	C4-A1	Mean
16	C4-A1	Mean power spectrum
17	C4-A1	RMS
18	C4-A1	Spectral entropy
19	F3-A2	Delta
20	F3-A2	Theta
21	F3-A2	Alpha
22	F3-A2	Beta
23	F3-A2	Zero crossings
24	F3-A2	Mean
25	F3-A2	Mean power spectrum
26	F3-A2	RMS
27	F3-A2	Spectral entropy
28	F4-A1	Delta
29	F4-A1	Theta
30	F4-A1	Alpha
31	F4-A1	Beta
32	F4-A1	Zero crossings
33	F4-A1	Mean
34	F4-A1	Mean power spectrum
35	F4-A1	RMS
36	F4-A1	Spectral entropy
37	O1-A2	Delta
38	O1-A2	Theta
39	O1-A2	Alpha
40	O1-A2	Beta
41	O1-A2	Zero crossings
42	O1-A2	Mean
43	O1-A2	Mean power spectrum
44	O1-A2	RMS
45	O1-A2	Spectral entropy
46	O2-A1	Delta
47	O2-A1	Theta
48	O2-A1	Alpha
49	O2-A1	Beta
50	O2-A1	Zero crossings
51	O2-A1	Mean
52	O2-A1	Mean power spectrum
53	O2-A1	RMS
54	O2-A1	Spectral entropy
55	Airflow	Apnea
56	EEG	Sleep stage
57	Abdo	RMS
58	Airflow	RMS
59	Position	Position
60	ECG	Zero crossings
61	ECG	Mean
62	ECG	Mean power spectrum
63	ECG	RMS
64	ECG	Spectral entropy
65	Chin EMG	Zero crossings
66	Chin EMG	Mean
67	Chin EMG	Mean power spectrum
68	Chin EMG	RMS
69	Chin EMG	Spectral entropy
70	HR	Heart rate
71	LOC	RMS
72	ROC	RMS
73	Sound	RMS
74	SpO2	Desaturation
75	SpO2	Lowest SpO2
76	SpO2	RMS
77	Thor	RMS

**Table 3:** Tabular results for 10-fold cross-validation for all folds and all model types (confusion matrix shows the classification of the cases in the test dataset). In confusion matrix, the columns denote the actual cases and the rows denote the predicted.

Fold number	Multilayer perceptron				*K*-nearest neighbor				Random forest				Logistic regression			
	Confusion matrix		Accuracy	RMSE	Confusion matrix		Accuracy	RMSE	Confusion matrix		Accuracy	RMSE	Confusion matrix		Accuracy	RMSE
1	378	55	0.8428	0.3562	432	38	0.9193	0.2841	445	40	0.9311	0.2584	406	67	0.8601	0.3352
	91	405			37	422			24	420			63	393		

2	367	56	0.8299	0.3676	433	41	0.9182	0.286	449	60	0.9139	0.2655	404	78	0.8461	0.3458
	102	404			35	420			20	400			65	382		

3	411	104	0.8256	0.3859	432	34	0.9236	0.2764	440	52	0.9128	0.2692	386	62	0.8439	0.3511
	58	356			37	426			29	408			83	398		

4	406	96	0.8288	0.3718	430	41	0.9139	0.2934	434	43	0.916	0.2665	385	67	0.8375	0.3561
	63	364			39	419			35	417			84	393		

5	378	56	0.8428	0.3619	426	32	0.9193	0.2841	428	53	0.8999	0.2821	410	87	0.8439	0.3512
	90	405			43	428			40	408			58	374		

6	386	79	0.8267	0.3768	422	36	0.9117	0.2971	427	50	0.902	0.2881	391	70	0.8407	0.3573
	82	382			46	425			41	411			78	390		

7	407	97	0.8297	0.3791	433	33	0.9267	0.2707	446	65	0.9063	0.2773	386	62	0.8448	0.3551
	61	363			35	427			22	395			82	398		

8	375	63	0.8319	0.3746	429	33	0.9224	0.2785	437	50	0.9127	0.2728	379	64	0.8351	0.3602
	93	397			39	427			31	410			89	396		

9	389	73	0.8362	0.3642	424	35	0.9149	0.2917	441	60	0.9063	0.2788	393	71	0.8427	0.3546
	79	387			44	425			27	400			75	389		

10	397	83	0.8341	0.367	431	40	0.917	0.288	426	54	0.8966	0.2812	390	68	0.8427	0.348
	71	377			37	420			42	406			78	392		

*Mean*			0.8329	0.3705			0.9187	0.285			0.9098	0.274			0.8437	0.3515

*Standard deviation*			0.0058	0.0084			0.0044	0.0077			0.0094	0.0086			0.0063	0.0068

## References

[1] American Academy of Sleep Medicine (2014). *International Classification of Sleep Disorders*.

[2] Karadeniz D., Ondze B., Besset A., Billiard M. (2000). Are periodic leg movements during sleep (PLMS) responsible for sleep disruption in insomnia patients?. *European Journal of Neurology*.

[3] The ASDA Atlas Task Force (1993). Recording and scoring leg movements. *Sleep*.

[4] Hornyak M., Feige B., Riemann D., Voderholzer U. (2006). Periodic leg movements in sleep and periodic limb movement disorder: prevalence, clinical significance and treatment. *Sleep Medicine Reviews*.

[5] David L. O., Dursun D. (2008). Advanced data mining techniques. *Data Mining Process*.

[6] American Electroencephalographic Society (1994). Guideline thirteen: guidelines for standard electrode position nomenclature. *Journal of Clinical Neurophysiology*.

[7] American Academy of Sleep Medicine (2007). *The AASM Manuel for The Scoring of Sleep and Associated Events*.

[8] Rechtshaffen A., Kales A. (1973). *A Manual of Standardized Terminology, Techniques and Scoring System for Sleep Stages in Human Objects*.

[9] Allen R. P., Picchietti D., Hening W. A. (2003). Restless legs syndrome: diagnostic criteria, special considerations, and epidemiology. A report from the restless legs syndrome diagnosis and epidemiology workshop at the National Institutes of Health. *Sleep Medicine*.

[10] Galar M., Fernández A., Barrenechea E., Bustince H., Herrera F. (2012). A review on ensembles for the class imbalance problem: bagging-, boosting-, and hybrid-based approaches. *IEEE Transactions on Systems, Man, and Cybernetics, Part C*.

[11] Umut İ. (2016). PSGMiner: a modular software for polysomnographic analysis. *Computers in Biology and Medicine*.

[12] O'Regan S., Faul S., Marnane W. (2013). Automatic detection of EEG artefacts arising from head movements using EEG and gyroscope signals. *Medical Engineering and Physics*.

[13] Wiggins M., Saad A., Litt B., Vachtsevanos G. (2008). Evolving a Bayesian classifier for ECG-based age classification in medical applications. *Applied Soft Computing*.

[14] Phinyomark A., Limsakul C., Phukpattaranont P. (2009). A novel feature extraction for robust EMG pattern recognition. *Journal of Computing*.

[15] Adrian E. D., Yamagiwa K. (1935). The origin of the berger rhythm. *Brain*.

[16] Scholl J. F., Agre J. R., Clare L. P., Gill M. C. A low power impulse signal classifier using the Haar wavelet transform.

[17] Oppenheim A. V., Schafer R. W., Horton M. J., Gilfillan A. (2013). Discrete-time signal processing. *Discreate-Time Signals and Systems*.

[18] Han J., Kamber M., Gray J. (2006). Data mining: concepts and techniques. *Data Mining Functionalities*.

[19] Haykin S. (1998). *Neural Networks: A Comprehensive Foundation*.

[20] Breiman L. (2001). *Machine Learning: Random Forest*.

[21] Hall P., Park B. U., Samworth R. J. (2008). Choice of neighbor order in nearest-neighbor classification. *The Annals of Statistics*.

[22] Hastie T., Tibshirani R., Friedman J. (2001). *The Elements of Statistical Learning*.

[23] Lavrac N. (1999). Selected techniques for data mining in medicine. *Artificial Intelligence in Medicine*.

[24] Omary Z., Mtenzi F. (2010). Machine learning approach to identifying the dataset threshold for the performance estimators in supervised learning. *International Journal for Infonomics*.

[25] Hu Q., Yu D., Xie Z. (2008). Neighborhood classifiers. *Expert Systems with Applications*.

[26] Çentik G. (2013). *Application of machine learning methods to polysomnography data [Ph.D. thesis]*.

